# The innate immune response of equine bronchial epithelial cells is altered by training

**DOI:** 10.1186/s13567-014-0126-3

**Published:** 2015-01-17

**Authors:** Linda Frellstedt, Philippe Gosset, Gwenola Kervoaze, Aymeric Hans, Christophe Desmet, Dimitri Pirottin, Fabrice Bureau, Pierre Lekeux, Tatiana Art

**Affiliations:** Center of Equine Sports Medicine, University of Liege, Liege, Belgium; CIRALE, École Nationale Vétérinaire de Maisons-Alfort, Goustranville, France; Institut Pasteur de Lille, Centre d’Infection et d’Immunité de Lille, Lille, France; Université Lille Nord de France, Lille, France; Centre National de la Recherche Scientifique, UMR8204 Lille, France; Institut National de la Santé et de la Recherche Médicale, U1019 Lille, France; Institut Fédératif de Recherche 142, Lille, France; Anses – Dozulé, Equine Pathology Laboratory, Virology Unit, Goustranville, France; Laboratory of Cellular and Molecular Immunology, GIGA-Research, University of Liege, Liege, Belgium

## Abstract

Respiratory diseases, including inflammatory airway disease (IAD), viral and bacterial infections, are common problems in exercising horses. The airway epithelium constitutes a major physical barrier against airborne infections and plays an essential role in the lung innate immune response mainly through toll-like receptor (TLR) activation. The aim of this study was to develop a model for the culture of equine bronchial epithelial cells (EBEC) in vitro and to explore EBEC innate immune responses in trained horses. Bronchial epithelial biopsies were taken from 6 adult horses during lower airway endoscopy. EBEC were grown in vitro by an explant method. The innate immune response of EBEC was evaluated in vitro by treatment with TLR ligands. TLR3 is the most strongly expressed TLR at the mRNA level in EBEC and stimulation of EBEC with Poly(I:C), an analog of viral dsRNA, triggers a strong secretion of IFN-β, TNF-α, IL-6 and CXCL8. We further evaluated the EBEC innate immune response in horses that underwent a 4-month-training program. While training had no effect on TLR mRNA expression in EBEC as well as in bronchial biopsies, it increased the production of IFN-β after stimulation with a TLR3 ligand and decreased the secretion of TNF-α and IL-6 after stimulation with a TLR2 and TLR3 ligand. These findings may be implicated in the increased risk for viral and bacterial infections observed in sport horses. Altogether, we report a successful model for the culture of EBEC that can be applied to the investigation of pathophysiologic conditions in longitudinal studies.

## Introduction

Respiratory diseases represent the second most common disorder requiring veterinary medical attention in adult horses [1]. They are also the second most common ailment in young racehorses [2,3] and result in huge economic losses to the racing industry. Both viral and bacterial infections as well as inflammatory conditions, such as Inflammatory Airway Disease (IAD), are mainly responsible for those respiratory diseases. Young horses are more susceptible to viral infection than older horses [4] and exercise potentiates this risk for viral infection [4,5]. Bacterial infections can occur as a primary disease or secondary to viral infections. IAD is a frequent problem in young sports horses, especially racehorses. Its prevalence varies from 7 to 70% depending on the geographic region and the horse population studied [6-9]. It is most frequently observed in 2 year olds [2,7,10-12] and has been associated with viral and/or bacterial infections [7,10-12]. The underlying pathophysiology of these respiratory diseases, especially the involvement of innate and adaptive immune mechanisms, is currently not well understood. However, intensive training has been reported to alter the innate immune response in the lung and the systemic circulation [13]. We have recently demonstrated that TLR3 mRNA expression as well as TNF-α and IFN-β production were down-regulated in equine pulmonary alveolar macrophages (PAM) from trained horses. On the contrary, TLR4 mRNA expression and cytokine secretion were amplified in blood monocytes.

The lung innate immune defense against pathogens also implicates the airway epithelium which strongly interacts with other resident cells and leukocytes. Indeed, the airway epithelium plays an essential role [14,15] firstly as a physical barrier between the external environment and the host [15,16] and secondly by modulating the inflammatory reaction and the response to pathogens [17]. The ciliated airway epithelium prevents colonization by inhaled bacteria through their physical removal by ciliary clearance and cough, through the presence of antimicrobial proteins in the mucus and through the recruitment of phagocytic cells [15,17]. In immune cells, a variety of pathogen recognition receptors (PRR), located on the surface or in intracellular compartments, interact with pathogen-associated molecular patterns (PAMP) and control the host defense mechanisms. Toll-like receptors (TLR) represent important signaling PRR that recognize PAMP [18,19]. TLR2 heterodimerizes with TLR1 or TLR6; TLR1/2 and TLR2/6 recognize molecular pattern specific for gram-positive bacteria. TLR3 mediates the response to double-stranded RNA, a marker of viral infection. TLR4 recognizes bacterial lipopolysaccharide (LPS) specific for gram-negative bacteria. TLR5 responds to flagellin which can be found on flagellated bacteria. TLR7/8 recognizes viral single-stranded RNA. Finally, TLR9 mediates the response to unmethylated DNA (CpG) which can be part of bacteria or viruses [18,19]. Once TLR are stimulated by PAMP, a downstream cascade is activated which results in the production of interferons (IFN), pro-inflammatory cytokines, chemokines, and cytotoxic activities [18,19]. Type I interferons are central to the early antiviral response of virus-infected cells [15] and interfere with viral replication, protein synthesis as well as early cell apoptosis. Activation of TLR is essential for an immediate protection against viral and bacterial infection. Epithelial cells express a large array of TLR and their activation leads to the secretion of a variety of cytokines (IFN, IL-6, TNF-α) and chemokines (CXCL8) [16]. CXCL8 is an essential chemotactic factor for neutrophils and its production is associated with viral, bacterial and fungal infections [16]. The mucosal response, including the innate response, aims to clear the sensed pathogens and to control secondary effects associated with neutrophils and their products [14]. Altogether, this underlines that TLR activation in the airway epithelium is important in order to tightly control the intensity and duration of this response in favor of preventing respiratory lesions [14].

In the interest of defining the physiopathologic role of bronchial epithelial cells in respiratory diseases in horses, ex vivo approaches are needed as previously reported in humans [20]. Few reports describe the use of bronchial epithelial cell culture in horses [21-27] and even fewer have used this technique for the study of equine diseases [21,22,27]. In order to perform this kind of study, we developed a method of culture for equine bronchial epithelial cells (EBEC) using bronchial biopsies collected from living animals. This method offers the possibility to perform repeated sampling. After the development phase of this protocol, we were able to obtain EBEC responsive to TLR ligands similarly to the airway epithelium, at least when considering the innate immune response. Based on this method, we analyzed the impact of intense training and acute exercise on the expression and function of TLR in EBEC to identify their role in equine respiratory diseases. Our results demonstrate that the expression of these receptors was not modified although the production of some cytokines in response to TLR ligands was altered. Using this new culture method, we were able to demonstrate some alteration in the function of EBEC in horses submitted to intense training.

## Materials and methods

### Horses

Six healthy Standardbred horses (3 mares and 3 geldings, 3 to 6 years old, 495.3 ± 55.2 kg) were used for the first part of the study to develop optimal EBEC culture conditions and EBEC responsiveness in vitro. All horses were determined to be healthy by physical examination, complete blood cell count, serum biochemistry, endoscopic evaluation of the respiratory tract and examination of the tracheal and bronchoalveolar lavage fluid (cytology, bacteriology and virology). We also investigated whether lower airway inflammation would be induced by the biopsy sampling procedure. Daily physical examinations were performed as well as lower airway endoscopy including tracheal washes and broncho-alveolar lavages on day 0 (prior to biopsy sampling) as well as 2 and 8 days after biopsy sampling. All vital parameters remained normal in all horses. None of the horses developed a cough or abnormal mucus secretion. The cytology results of the tracheal washes and broncho-alveolar lavages remained normal in all horses (data not shown). During lower airway endoscopy, we did not observe any local inflammation (reddening, swelling) at the biopsy sites; whereas a fast healing process was detected. On day 8, it was often not possible to identify the biopsy site anymore.

Once the EBEC culture conditions had been defined, eight healthy untrained (UT) Standardbred horses (5 mares and 3 geldings, 2.5 ± 0.5 years, 450 ± 45 kg) were enrolled for the second part of our study. All horses were determined to be healthy by physical examination, complete blood cell count, serum biochemistry, endoscopic evaluation of the respiratory tract and examination of the tracheal and bronchoalveolar lavage fluid (cytology, bacteriology and virology). The horses had been vaccinated (equine herpesvirus 1 and 4, equine influenza, tetanus) and dewormed and were kept in a grass pasture prior to starting the study. During the training period, the horses were stabled individually in well-ventilated stalls on wood shavings. They were fed grass silage and grain. In addition, they had daily access to a large paddock for a minimum of 4 h.

Our protocols were approved by the Animal Ethics Committee of the National Veterinary School of Alfort (agreement number: 13/12/11-9) and the Animal Ethics Committee of the University of Liege (agreement number: 13–1510).

### Training protocol

Prior to the start of the study, the horses were kept in a grass pasture for 3 months (untrained = UT). This period allowed the horses to get accustomed to the environment. The horses started with 8 weeks of moderate training (MT), meaning they were exercised on a sand track three times a week. Each training session consisted of a six minute warm-up at 5.5 m/s, then trotting at progressively increasing speeds (7.0 to 9.7 m/s) for 20 min, followed by a recovery period of 3 min at 5.5 m/s. This was followed by the intensive training (IT) period of 8 weeks. They were exercised five times a week. Three sessions per week were performed as during the moderate training period at gradually increasing speeds up to their individual maximum speeds for 20 min. Two sessions per week consisted of interval (“sprint”) training at maximal speeds. During these sessions, the horses underwent a warm-up of 15 min at 5.5 m/s, followed by 7 min at 7.0 m/s, then during a period of 10 min the horses maintained speeds of 9.7 to 11.0 m/s with the intercalated intervals (3 × 90 s) at maximal speeds (11.0 to 13.4 m/s), finishing with a recovery period of 3 min at 5.5 m/s. Heart rates, distance and speed were monitored continuously during each training session using a GPS on-board system (Equine CS600X Trotting, Polar Electro France SAS).

### Standardized exercise test (SET)

The horses underwent a SET on a treadmill until fatigue prior to and at the end of the training period. The SET was performed as previously published [9]. A fourth step was performed at 11.0 m/s until fatigue.

### Reagents

All reagents were purchased from Life Technologies, Paisley, UK unless otherwise stated.

### Sample collection

The horses were sedated with detomidine (0.002 mg/kg) and butorphanol (0.005 mg/kg). Lower airway endoscopy was performed (Karl Storz, Utrecht, Netherlands). Bronchial biopsies (2 × 2 mm) were collected between the 1^st^ and 3^rd^ generation bronchi with biopsy forceps (Pentax, Aartselaar, Belgium). The bronchial biopsies were placed in cold DMEM/F-12 medium (Lonza, Verviers, Belgium) supplemented with penicillin (100 IU/mL), streptomycin (100 μg/mL) and amphotericin (0.25 μg/mL). The samples were kept at 4 °C and processed for cell culture within 2 h of collection. Concurrently, bronchial biopsies were placed in RNAlater® and stored at −20 °C until analysis for TLR and cytokine mRNA expression.

In the second part of the study, bronchial biopsies were collected at rest (48 h prior to the SET) between the 1^st^ and 3^rd^ generation bronchi of the left lung and post-exercise (24 h after the SET) between the 1^st^ and 3^rd^ bronchi of the right lung from horses in the untrained and trained condition.

### Cell culture

In preliminary experiments, we tested a number of different culture conditions in order to identify optimal culture conditions for EBEC. Tissue culture plates were coated with human placental collagen type I, human placental collagen type IV (Sigma-Aldrich, St. Louis, MO, USA), calf skin collagen G (Biochrom, Merck Millipore, Darmstadt, Germany) as well as a mix of human fibronectin with bovine serum albumin (BSA) (Merck Millipore, Darmstadt, Germany) and collagens. We also used different culture media: BEGM (Promocell, Heidelberg, Germany), LHC-8 medium, DMEM/F-12 with 2% ultroser, a mix of LHC-8 and DMEM/F-12, a mix of BEGM and DMEM/F-12, with and without growth factors (epithelial growth factor, bovine pituitary extract).

After collection, the bronchial biopsies were washed three times in DMEM/F-12 medium. The biopsies were then placed on coated tissue culture plates (6-well plates, NUNC, Thermo Fisher Scientific, Erembodegem-Aalst, Belgium) in a minimal volume of medium supplemented with penicillin (100 IU/mL), streptomycin (100 μg/mL) and amphotericin (0.25 μg/mL) and placed in the incubator at 37 °C with 5% CO_2_ for 24–48 h in order to favor the adherence of the biopsy. For the second part of the study, bronchial biopsies were placed on collagen IV coated plates in DMEM/F-12 medium supplemented with 2% ultroser (Pall Life Science, Cergy-Saint-Christophe, France), penicillin (100 IU/mL), streptomycin (100 μg/mL) and amphotericin (0.25 μg/mL). The medium was changed every 2 to 3 days until the cell cultures reached confluency (three to four weeks). The epithelial cell cultures were assessed daily under the light microscope for confirmation of their growth pattern (polygonal shape), and to rule out possible contamination with fibroblasts. We obtained 5 × 10^5^ to 1 × 10^6^ cells from a single explant. Once cultures were confluent, the EBEC were detached with trypsin, placed into DMEM/F-12 medium with 10% fetal bovine serum (FBS; Fisher Scientific, Aalst, Belgium), penicillin (100 IU/mL), streptomycin (100 μg/mL) and amphotericin (0.25 μg/mL). The cells were centrifuged, placed in DMEM/F-12 medium with 2% ultroser and then transferred into 24-well tissue culture plates (1 × 10^5^ cells per well) for cell proliferation and subsequent stimulation with TLR ligands. One aliquot of 1 × 10^5^ EBEC was used for flow cytometry systematically at each passage of cells from each horse. Once confluency was reached again (five to seven days), EBEC were stimulated with TLR2/6, TLR3, TLR4, TLR7/8 and TLR9 ligands (FSL- 100 ng/mL; Poly(I;C)- 50 μg/mL (CAYLA, Toulouse, France); LPS- 100 ng/mL (Sigma-Aldrich, Saint-Louis, MO, USA); Gardiquimod- 1 μg/mL (CAYLA, Toulouse, France); CpG- 1 μg/mL (composition: 5′-tccatgacgttcctgatgct-3′, class B CpG, Hycult Biotech, Uden, Netherlands), respectively. Systematically, EBEC from one well of non-stimulated cells were used for flow cytometry and EBEC from a second well of non-stimulated and stimulated cells were placed in Trizol® for the assessment of TLR mRNA expression. Only cells from the first passage, that were confirmed to be epithelial cells of excellent purity (at least 95% cytokeratin positive and negative for vimentin), were used for the evaluation of TLR mRNA expression, TLR stimulation and the recovery of supernatants for cytokine secretion.

Primary fibroblasts were grown from deeper bronchial biopsy tissue. They had an accelerated growth rate and reached confluency within 7–10 days.

### Light microscopy

The primary EBEC and fibroblast cultures were monitored daily under a phase contrast inverted microscope (Olympus, Tokyo, Japan). The morphology of the EBEC was documented and fibroblast contamination of the EBEC cultures was ruled out.

### Flow cytometry

To confirm the EBEC epithelial character, EBEC were assessed systematically during their transfer (first passage) and at the time of stimulation with TLR ligands. EBEC were labeled with anti-cytokeratin antibodies (mouse anti-human cytokeratin, clone MNF116, Dako, Hamburg, Germany). Anti-vimentin antibodies (mouse anti-bovine vimentin, clone Vim3B4, Dako, Hamburg, Germany) were used to confirm the absence of fibroblast contamination. Donkey anti-mouse IgG labeled with Alexa Fluor® 488 dye were used as secondary antibodies.

Once confluent, EBEC were detached from tissue culture plates with trypsin, placed in DMEM/F-12 medium with 10% FBS and cytocentrifuged. The cells were fixed in 1% formaldehyde, washed twice in 0.1% saponin to allow for cell permeabilization and then incubated with anti-cytokeratin or anti-vimentin antibodies for 30 min at 4 °C. The cells were again washed in 0.1% saponin and were incubated with the secondary antibodies for 30 min at 4 °C. Then they were washed twice in PBS and placed in 0.25% formaldehyde solution and analyzed within 24 h. Flow cytometry analysis was performed on a BD Flowcytometer (BD FACSCanto II, BD Biosciences, Erembodegem, Belgium). In all conditions, negative controls were prepared by omitting primary antibodies. The primary fibroblasts were stained with the same antibodies in order to confirm their mesenchymal origin.

### Cytokine measurement by ELISA

Cell culture supernatants were assayed for TNF-α (R&D Systems, Abingdon, UK), IFN-β, IL-6 (USCN Life Science Inc., Wuhan, China) and CXCL8 (Kingfisher Biotech Inc., Breda, Netherlands) concentrations with commercially available equine ELISA kits.

### Muscle tissue

Microbiopsies of the gluteus medius muscle were obtained as previously described [28]. This tissue served as calibrator/control tissue for the TLR expression as previously described [28] since muscle tissue is known to have the lowest TLR expression within the body tissues [29].

### RNA isolation, cDNA synthesis and real-time PCR

RNA was isolated from bronchial biopsies, muscle biopsies and cultured EBEC with Trizol® reagent as described by the manufacturer. Contaminating DNA was eliminated using the Turbo DNA-free kit™. Concentration and quality of RNA were assessed by UV spectrophotometry (Nanodrop® ND2000, Thermo Fisher Scientific, Aalst, Belgium) and a 2100 Bioanalyzer (Agilent Technologies, Waldbronn, Germany). UV spectrophotometry allowed the determination of the ratio of absorbance at 260 nm and 280 nm. This ratio is used to assess the purity of RNA. A ratio of 1.8-2.0 was accepted as indication of “good quality” RNA. The quality of the RNA was further assessed by a 2100 Bioanalyzer. Only RNA samples with an RNA integrity number (RIN) ≥ 9 and a 28S:18S ratio of > 2:1 qualified as “good quality” RNA and were used for real-time PCR. cDNA were generated as previously described [28]. Gene expression of TLR1-9, fibroblast growth factor-1 (FGF-1), fibroblast specific protein-1 (FSP-1), E-cadherin, keratin19, IFN-β, TNF-α, IL-6 and CXCL8 was quantified by real-time PCR using Absolute Blue qPCR SYBR Green Mix (Fisher Scientific, Aalst, Belgium) on an Applied Biosystems 7900 HT thermocycler (Applied Biosystems, Carlsbad, CA, USA). Equine gene specific primers were designed from available published sequences [13,26,30,31] or designed in silico (Table 1). Specificity of PCR was confirmed by melting curve analysis, agarose gel electrophoresis and the absence of signals in RT minus RT reactions. All primers designed for this study were located in single exons except the primer for CXCL8 (2 separate exons). The absence of signals in RT minus RT reactions and the fact that all primers, except for one, were located in single exons confirm the absence of DNA contamination. Real-time quantitative PCR data for TLR1-9, FGF-1, FSP-1, E-cadherin, keratin19, IFN-β, TNF-α, IL-6 and CXCL8 were normalized to the geometric mean of three housekeeping genes (PGK1 (phosphoglycerokinase 1), PPIA (cyclophilin A), RPL0 (ribosomal protein L0)) using qbase + software (Biogazelle, Zwijnaarde, Belgium). Muscle tissue was used as a calibrator for the characterization of TLR mRNA expression in bronchial biopsies and EBEC. The dCT results were compared to the calibrator samples to derive the relative quantities.

**Table 1:** **Primer sequences used for real-time PCR analysis of mRNA expression of target and housekeeping genes**

**Name**	**Primer sequences forward/reverse (5′ to 3′)**	**Product (bp)**	**Exon**	**Reference**
PGK1	CGTCAAAGACCTGATGTCCA	98	1	In silico designed
	TCTTGGCATGCTCATCAAAC			
PPIA	TGGGGAGAAATTTGATGATGA	116	1	In silico designed
	TGGCAGTGCAGATGAAGAAC			
RPL0	CCAAGGTTGAAGCAAAGGAA	99	1	[13]
	AAAGCTGGCTGAATTGGTTG			
FGF-1	AGAAGAATGGGAGCTGCAAA	138	1	In silico designed
	GGACTCCTTCAGGCTCCTCT			
FSP-1	GCCCTGGATGTGATGGTATC	93	1	In silico designed
	CAGCAGCTCCTTTAGCTCTGA			
E-cadherin	ATCCGCAGCCTCATATCATC	125	1	In silico designed
	CCCACCTGGGTCATTGTACT			
Keratin19	GAAGGAGACCATGCAGAACC	120	1	In silico designed
	CCCTGCTTCAGGTACCAGTC			
IFN-beta	CCCCGAGGACACAATGAACT	81	1	In silico designed
	ACCAATGCAGCATCCTCCTT			
TNF-alpha	AAGGACATCATGAGCACTGAA	80	1	In silico designed
	GGGCCCCCTGCCTTCT			
IL-6	CCTGGTGATGGCTACTGCTT	102	1	In silico designed
	TGCTGTTTGGTTTTGTCTGC			
CXCL8	TTGGCCGTCTTCCTGCTTT	101	2 Intron (959 bp)	In silico designed
	GGTTTGGAGTGCGTCTTGATG			

### Statistical analysis

TNF-α, IFN-β, IL-6 and CXCL8 concentrations as well as relative gene expression data were analyzed by a global linear mixed model (SAS, Cary, NC, USA). A general linear mixed model was used for data analysis because it combines a random effect (horses) and fixed effects (training, strenuous exercise, treatment with TLR ligands). The mixed model allows considering the random nature of the horses sample and accordingly, correcting for potential individual differences, while modeling the other (fixed) effects of interest in our study. In such a multifactorial and unbalanced design, results were reported as least square means (LSmeans) ± associated SE instead of simple means in order to correct each factor for possible contamination due to the other factors of the model. Differences were considered to be significant when p value was less than 0.05.

## Results

### Growth and characterization of EBEC in vitro

**Figure 1:**
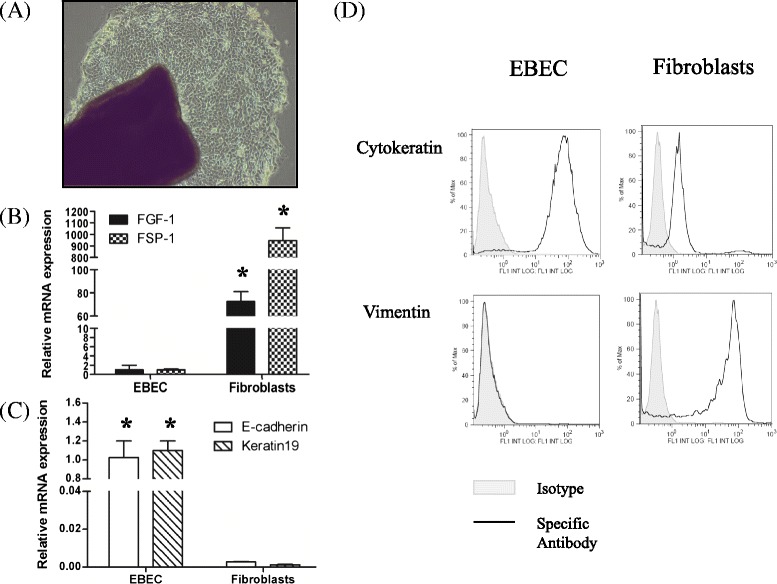
**Characterization of equine bronchial epithelial cells (EBEC). (A)** Morphological appearance of EBEC cultures 7 days after plating (phase contrast microscopy; objective magnification: x4). The biopsy is situated on the left bottom corner of the image. The biopsy is surrounded by growing EBEC. Note the polygonal shape of EBEC. **(B)** Relative mRNA expression of Fibroblast growth factor-1 (FGF-1) and Fibroblast specific protein-1 (FSP-1) in EBEC and fibroblasts. Significant differences (*P* < 0.05) in mRNA expression are identified with * for differences between EBEC and fibroblasts. **(C)** Relative mRNA expression of E-cadherin and Keratin19 in EBEC and fibroblasts. Significant differences (*P* < 0.05) in mRNA expression are identified with * for differences between EBEC and fibroblasts. **(D)** Flow cytometry analysis of in vitro cultured EBEC and fibroblasts for the epithelial cell marker cytokeratin and the mesenchymal cell marker vimentin. **(C-D)** EBEC were assessed at their first passage (4–6 weeks of culture). Fibroblasts were assessed at their first passage (7–10 days of culture). The phenotype and purity of EBEC were maintained during the study.

**Figure 2:**
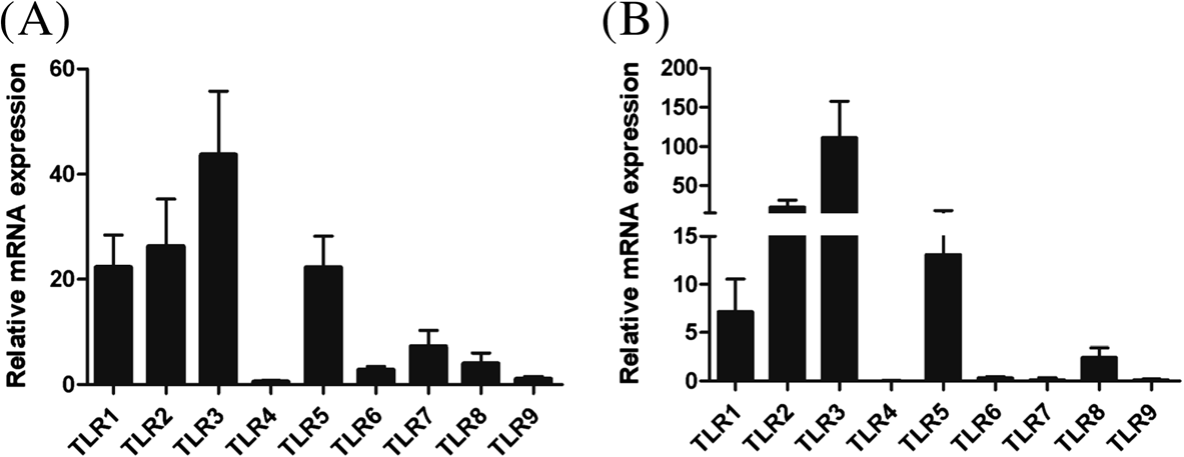
**Relative mRNA expression of TLR1-9 in bronchial biopsies (A) and EBEC (B).** Muscle tissue is used as a calibrator for TLR mRNA expression (muscle tissue = 1). Note that the profile of mRNA expression of TLR1-9 in bronchial biopsies and EBEC is similar, although the magnitude of TLR mRNA expression is amplified after in vitro culture of 4–6 weeks duration (first passage).

**Figure 3:**
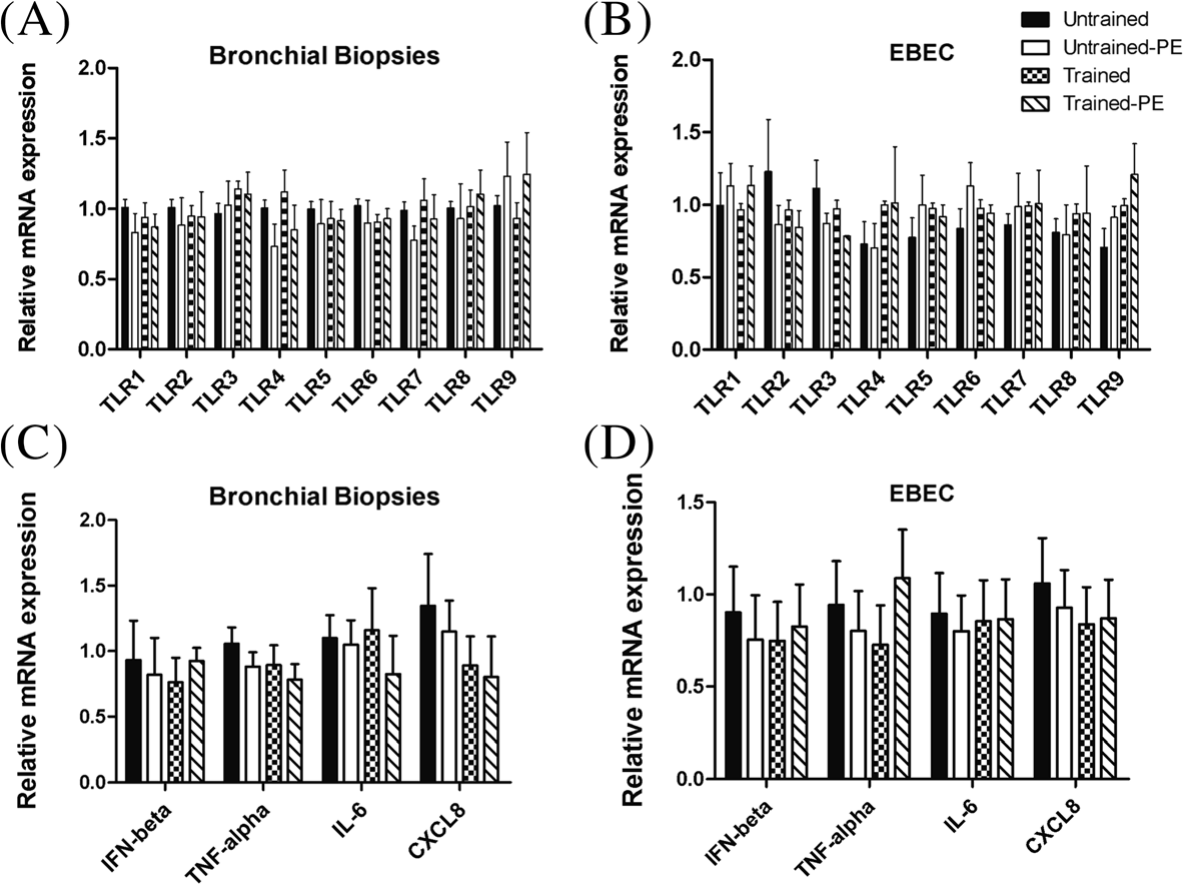
**Relative mRNA expression of TLR1-9 and four cytokines in bronchial biopsies and cultured EBEC from untrained and trained horses.** The relative mRNA expression of TLR1-9 **(A,B)** and four cytokines (IFN-β, TNF-α, IL-6, CXCL8) **(C,D)** was evaluated directly in bronchial biopsies **(A,C)** and in non-stimulated cultures of EBEC **(B,D)** obtained from biopsies collected from untrained and trained horses, at rest as well as after an acute exercise (PE = post-exercise). No significant differences were observed.

Homogenous cell populations consisting of non-ciliated EBEC were cultured successfully using an explant method. Our preliminary tests showed that the EBEC grew better on collagen IV coated plates in DMEM/F-12 medium with 2% ultroser (Table 2). The supplementation with growth factors had no effect on the growth of EBEC in our model. The cells had a polygonal shape which is typical for the morphology of epithelial cells (Figure 1A). The epithelial cell character was confirmed by positive staining for cytokeratin (Figure 1D) during each step of the EBEC culture (passage of EBEC and at time of stimulation) and during each phase of the study (untrained and trained horses). Fibroblast contamination represents a common problem in epithelial cell culture. Our data revealed that EBEC are negative for vimentin in comparison with cultures of fibroblasts (Figure 1D). Fibroblasts strongly expressed mRNA encoding for FGF-1 and FSP-1, whereas these mRNA were expressed about 100–1000 times less in EBEC (Figure 1B). In contrast, EBEC strongly expressed E-cadherin and keratin19 (Figure 1C). The EBEC phenotype was further confirmed by TLR mRNA expression, particularly of TLR3 (as discussed in the following paragraph). Figure 2 shows that EBEC have a similar pattern of TLR mRNA expression as the bronchial epithelial biopsy tissue itself. Furthermore, we show that the TLR mRNA expression was neither altered by our culture technique (Figure 2) nor by training of the horses (Figure 3B). Our results indicate that our EBEC cultures had excellent purity, and that their phenotype was maintained during the study.

**Table 2:** **Comparison of different cell culture conditions for the in vitro culture of EBEC**

**Matrix coating medium**	**Collagen I**	**Collagen IV**	**Collagen I + collagen IV**	**Collagen G**	**Fibronectin + BSA + collagen I**	**Fibronectin + BSA + collagen IV**
**BEGM**	**(+)**	**(+)**	**(+)**	**(+)**	**-**	**-**
**LHC-8**	**+(+)**	**++**	**+**	**+**	**-**	**-**
**DMEM/F12 + 2% Ultroser**	**+(+)**	**+++**	**++**	**+(+)**	**-**	**-**
**DMEM/F12 + 10% FBS**	**-**	**-**	**-**	**-**	**-**	**-**
**50% BEGM +50% DMEM/F12**	**+**	**+**	**+**	**+**	**-**	**-**
**50% LHC-8 + 50% DMEM/F12**	**+**	**(+)**	**+**	**(+)**	**-**	**-**

No growth - ; limited growth for 10 days only (+) ; growth during 10 days only + ; growth during the entire culture period ++/+++.

### TLR expression in bronchial biopsies and in cultures of EBEC

To estimate the relevance of our culture model, we compared the expression of TLR in bronchial biopsies and EBEC. As previously described in EBEC [32,33], TLR3 was the most strongly expressed TLR at the mRNA level in bronchial biopsies (Figure 2A) as well as in EBEC cultured in vitro (Figure 2B), followed by TLR2, TLR5 and TLR1. However, the magnitude of TLR mRNA expression was amplified during in vitro culture. In both types of samples, the expression of TLR4, TLR6, TLR7 and TLR9 was low and nearly undetectable. Thus, we conclude that the pattern of TLR mRNA expression was similar between bronchial biopsies and EBEC.

### Cytokine mRNA expression in EBEC stimulated in vitro with TLR ligands

**Figure 4:**
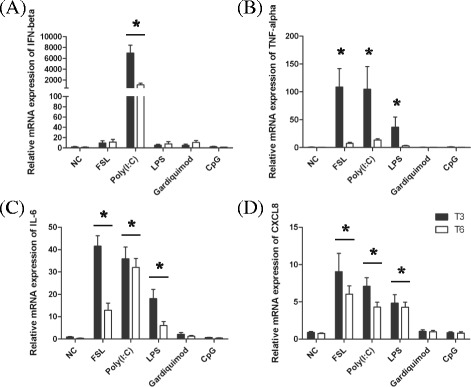
**Relative mRNA expression of IFN-β, TNF-α, IL-6 and CXCL8 after stimulation of EBEC with TLR ligands.** EBEC were stimulated with FSL, Poly(I:C), LPS, Gardiquimod and CpG. The mRNA expression of IFN-β **(A)**, TNF-α **(B)**, IL-6 **(C)** and CXCL8 **(D)** was evaluated after 3 and 6 h of incubation. Significant differences (*P* < 0.05) in mRNA expression are identified with * for differences between non-stimulated cells (NC) and cells stimulated with TLR ligands.

To analyze the responsiveness of EBEC, we evaluated the cytokine production after stimulation with TLR ligands. The IFN-β mRNA expression was significantly increased only by treatment with Poly(I:C), a TLR3 ligand, after 3 (*P* < 0.0001) and 6 (*P* < 0.005) hours of incubation (Figure 4A). Treatment of EBEC with FSL (*P* < 0.0003), LPS (*P* < 0.035) and Poly(I:C) (*P* < 0.0004) induced a significant increase in mRNA expression of TNF-α (Figure 4B) after 3 hours of incubation. The mRNA expression of IL-6 (Figure 4C) and CXCL8 (Figure 4D) was significantly upregulated after 3 and 6 h of incubation when the EBEC had been treated with FSL (*P* < 0.006; *P* < 0.0001), Poly(I:C) (*P* < 0.0001; *P* < 0.0006) and LPS (*P* < 0.02; *P* < 0.0013). Treatment of EBEC with Gardiquimod and CpG did not induce the mRNA expression of these cytokines (Figures 4A-D). While Gardiquimod and CpG represent appropriate TLR7/8 and TLR9 ligands, we conclude that the low expression of TLR7/8 and TLR9 in EBEC accounts for the non-responsiveness of EBEC to these ligands.

### Cytokine production in EBEC stimulated in vitro with TLR ligands

**Figure 5:**
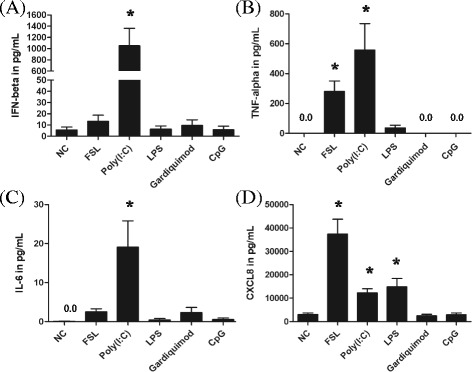
**Production of IFN-β, TNF-α, IL-6 and CXCL8 after stimulation of EBEC with TLR ligands.** EBEC were stimulated with FSL, Poly(I:C), LPS, Gardiquimod and CpG. The protein secretion of IFN-β **(A)**, TNF-α **(B)**, IL-6 **(C)** and CXCL8 **(D)** after 6 h incubation of EBEC in medium alone (NC) and after treatment with TLR ligands (FSL, Poly(I:C), LPS, Gardiquimod, CpG). Significant differences (*P* < 0.05) are identified with * for differences between non-treated cells (NC) and cells treated with TLR ligands.

Because the mRNA expression of cytokines is sometimes not correlated with the protein secretion in certain cells, we decided to examine the cytokine production in EBEC. Since the peak in cytokine mRNA expression was observed after 3 h of incubation, the cytokine production was measured after 6 h of stimulation. As previously reported [34], non-stimulated EBEC produced almost non-detectable levels of IFN-β, TNF-α and IL-6 but had an important baseline secretion of CXCL8 (Figures 5A-D). Only stimulation with Poly(I:C) significantly increased the production of IFN-β (*P* < 0.0001) (Figure 5A) and IL-6 (*P* < 0.0001) (Figure 5C). TNF-α secretion (Figure 5B) was significantly induced by treatment with FSL (*P* < 0.0003) and Poly(I:C) (*P* < 0.0001). CXCL8 secretion was significantly amplified by stimulation of EBEC with FSL (*P* < 0.0001), Poly(I:C) (*P* < 0.0005) and LPS (*P* < 0.0005) (Figure 5D). Treatment with Gardiquimod and CpG had no or limited effects on cytokine secretion (Figures 5A-D).

Since EBEC express low or undetectable levels of TLR7/8 and TLR9 and they did not respond to stimulation with a TLR7/8-ligand (Gardiquimod) or TLR9-ligand (CpG), for the second part of the study we focused on TLR2/6, TLR3, TLR4 in order to account for the most common respiratory pathogens in horses [13].

### Training has no effect on TLR or cytokine mRNA expression in bronchial biopsies and non-stimulated EBEC

The TLR mRNA expression was examined directly in bronchial biopsy tissue and in non-stimulated EBEC cultured in vitro. Training or acute strenuous exercise had no effect on the TLR mRNA expression in either of them (Figure 3A and B). We also evaluated cytokine mRNA expression directly in bronchial biopsies and in non-stimulated EBEC in order to assess if training or acute exercise had an immediate effect on the baseline cytokine mRNA expression. No significant differences were observed after training or acute strenuous exercise regarding the mRNA expression of IFN-β, TNF-α, IL-6 or CXCL8 (Figure 3C and D).

### Training alters the cytokine response to TLR ligands in EBEC

**Figure 6:**
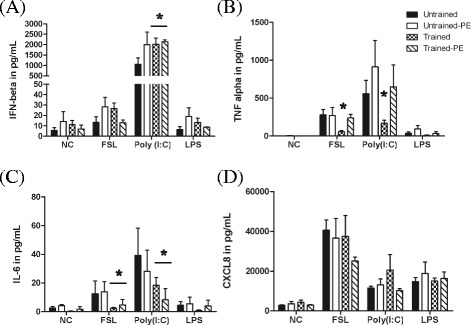
**Ex vivo production of IFN-β, TNF-α, IL-6 and CXCL8 in EBEC from untrained and trained horses.** EBEC were obtained from biopsies collected from untrained and trained horses, at rest as well as after an acute exercise (PE = post-effort). EBEC were cultured in vitro and stimulated with FSL, Poly(I:C) and LPS. We report the secretion of IFN-β **(A)**, TNF-α **(B)**, IL-6 **(C)** and CXCL8 **(D)** after 6 h of incubation in EBEC in medium alone (NC) and activated cells. Significant differences (*P* < 0.05) are identified with * for differences from EBEC in untrained horses.

In order to assess the effect of training and/or acute exercise on the innate immune response, the cultured EBEC were treated with TLR2/6, TLR3 and TLR4 agonists. EBEC collected after an acute exercise responded to TLR ligands in a similar manner as the controls (Figures 6A-D). In contrast, the EBEC obtained from trained horses showed an increased secretion of IFN-β (*P* < 0.0003) (Figure 6A) and a lower production of TNF-α and IL-6 after treatment with FSL (*P* < 0.05; *P* < 0.05) and Poly(I:C) (*P* < 0.0003; *P* < 0.01) (Figure 6B and C). The TNF-α secretion of FSL- and Poly(I:C)-stimulated EBEC from trained horses after an acute exercise was restored to a level similar to untrained horses, whereas the IL-6 production was still strongly decreased (Figure 6C). The response to LPS for IL-6 and CXCL8 was not modified. Training and acute exercise had no effect on the CXCL8 secretion induced by TLR ligands (Figure 6D).

## Discussion

We report the successful culture of EBEC in vitro from bronchial biopsies obtained in live horses. Our model allows us to culture EBEC from small biopsies obtained from the bronchial epithelium. EBEC do not cause any lower airway inflammation and do not present a risk for the horses. The in vitro cultured EBEC are responsive to TLR activation, therefore, they facilitate the investigation of pathophysiologic conditions in longitudinal studies as described in this work.

Previously, other authors have reported the successful culture of airway epithelial cells in horses [21-26]. The use of FBS in their culture medium was described and appeared to favor tight junction formation in their studies [25]. As shown by others [35-39], we found that FBS increased the contamination of EBEC cultures with fibroblasts, accelerated fibroblast growth and indirectly limited EBEC growth. Specific epithelial cell culture media are commonly used in human and veterinary research and were tested in our study. These defined media as well as specific growth factors did not support the growth of EBEC in our conditions. We hypothesize that the growth of EBEC from bronchial biopsies is even more fragile and sensitive than primary cell culture of airway epithelial cells recovered by digestion of bronchial epithelial tissue in which case large quantities of cells are made available. We show that DMEM/F-12 medium supplemented with a serum substitute (Ultroser G®) appeared to be the most appropriate medium for the culture of EBEC as we previously described in human BEC [20]. In this condition, we detected no detectable contamination by fibroblasts in our cultures. Moreover, the phenotype and cell purity were maintained during the culture as confirmed in a previous report using the same methodology in clinical immunology [20].

As expected, TLR3 was the most importantly expressed TLR at the mRNA level in EBEC, followed by TLR2, TLR5 and TLR1. TLR4 and TLR6-9 were expressed at very low levels. TLR1-6 proved to be functional in our study with Poly(I:C) being the most efficient stimuli as a TLR3 agonist. These findings were in agreement with other studies [32,33,40]. Previously, Quintana et al. [26] described a model for the culture of equine respiratory epithelial cells and examined the expression of TLR1-4 and TLR6-10 mRNA. They reported decreased mRNA expression of TLR8-10 after 4 weeks of in vitro culture. In our model, the profile of TLR mRNA expression remained similar in EBEC when compared to bronchial biopsies although the magnitude of TLR mRNA expression was amplified during in vitro culture. Nonetheless, TLR3 was still the most importantly expressed TLR (also with the highest responsiveness) after several weeks of culture. The stimulation of TLR3 activates the transcription factors NF-кB and interferon regulatory factor 3 (IRF3) which significantly increase IFN-β, TNF-α, IL-6 and CXCL8 production confirming the importance of EBEC in the anti-viral defense mechanisms of the lung [41]. Type-I IFN will inhibit viral replication, whereas the proinflammatory cytokines (TNF-α, IL-6) and chemokines (CXCL8) play important roles in immune cell recruitment. The mRNA expression of TLR4 appears to be lower in EBEC compared to its expression in human bronchial epithelial cells (HBEC) [32,33,40]. This could represent a protective mechanism in EBEC since horses are continuously exposed to large quantities of dust and organic particulates (shavings, straw, hay) that contain endotoxin [21]. Nevertheless, LPS stimulation induced the secretion of IL-6 and at a lower level TNF-α underlining the functionality of TLR4. Furthermore, Ainsworth et al. reported an upregulation of TLR4 in bronchial epithelial cells of horses with recurrent airway obstruction (RAO) which renders them more sensitive to such endotoxin exposure. We also report that TLR7-9 are not functional in EBEC as confirmed by the treatment with TLR7-9 agonists. This was in agreement with previous publications in HBEC [33,34].

The effect of training and acute exercise on TLR-associated airway epithelial cell function has not been reported previously. Several researchers have examined the effect of exercising in a cold environment on the airway epithelium. Bronchial epithelial cell damage, apoptosis, loss of ciliated epithelial cells and airway remodeling have been described in sled dogs [42] and horses [43]. In this study, neither training nor acute strenuous exercise had an effect on the mRNA expression at baseline of TLR or cytokines in bronchial biopsy tissue and EBEC. Our data underline that even though some damage can be observed in airway epithelium after exercise, this was not associated with airway inflammation and modulation of TLR mRNA expression. Only the exposure to TLR agonists, which mimicked experimental bacterial/viral infection, revealed altered EBEC function in cells from trained horses. Intense training but not acute exercise impaired the secretion of TNF-α and IL-6 only in response to FSL and Poly(I:C). FSL mimics infection with gram + bacteria, whereas Poly(I:C) mimics viral infection, thereby representing the most common equine pulmonary pathogens. The down-regulated production of pro-inflammatory cytokines could be caused by secretion of anti-inflammatory cytokines as suggested by others [44]. We did not evaluate anti-inflammatory cytokines (IL-10, IL-1ra, TGF-β) in this study, and therefore cannot argue their importance here. The decreased TNF-α secretion might also suggest a defect in cell recruitment by EBEC in trained horses. TNF-α is one of the first cytokines produced after stimulation with TLR ligands, exposure to pathogens or other irritants. It then stimulates the secretion of other cytokines and chemokines (CCL2, CCL3) that result in the recruitment of monocytes and their differentiation into dendritic cells and tissue macrophages. This alteration in cell recruitment might play an important role in limiting the lung defense mechanism and therefore, represent an essential mechanism in organ-specific regulation of immunity [45,46]. In contrast, IFN-β production by EBEC was increased after training. We recently described decreased TLR3 mRNA expression and decreased IFN-β production in equine PAM after training [13]. The increased IFN-β secretion by EBEC might reflect a compensatory mechanism for the low pulmonary IFN-β levels in trained horses. Altogether, the decreased type I IFN and cytokine secretion in the lung from trained horses might favor the development of viral infection and thereby, might increase secondary bacterial infection in the lung. As already mentioned in our previous work [13], our data show that the type and the intensity of the exercise (acute exercise/training) as well as the environmental conditions play an important role in the innate immune defense.

In conclusion, we report a successful model for the culture of EBEC in vitro that can be applied to the investigation of pathophysiologic conditions in longitudinal studies. These EBEC present a profile of expression and function of TLR similar to what we can find in bronchial biopsies and in other cell culture models. Training altered the IFN-β, TNF-α and IL-6 secretion in response to TLR2 and TLR3 ligands in EBEC and, therefore, may have important effects on immune cell recruitment in exercising horses. Altogether, this mechanism may be implicated in the increased risk for viral and bacterial infections observed in sport horses.

## Abbreviations

BSA: Bovine serum albumin
CpG: Unmethylated CpG dinucleotides
EBEC: Equine bronchial epithelial cells
FBS: Fetal bovine serum
FGF-1: Fibroblast growth factor-1
FSL: Synthetic diacylated lipoprotein
FSP-1: Fibroblast specific protein-1
HBEC: Human bronchial epithelial cells
IAD: Inflammatory airway disease
IFN: Interferon
IT: Intensive training
LPS: Lipopolysaccharide
MT: Moderate training
PAM: Pulmonary alveolar macrophages
PAMP: Pathogen-associated molecular pattern
Poly(I:C): Polyinosine-polycytidadylic acid
PRR: Pathogen recognition receptor
RAO: Recurrent airway obstruction
SET: Standardized exercise test
TLR: Toll-like receptor
UT: Untrained
